# Screening of Domestic Cats from North-Eastern Hungary for *Hepatozoon felis* and *Cytauxzoon europaeus* That Cause Infections in Local Wildcat Populations

**DOI:** 10.3390/pathogens12050656

**Published:** 2023-04-28

**Authors:** Barbara Tuska-Szalay, Sándor A. Boldogh, Róbert Farkas, Luca Rompos, Nóra Takács, Viktor Beresnyák, Ádám Izsó, Jenő Kontschán, József Lanszki, Sándor Hornok

**Affiliations:** 1 Department of Parasitology and Zoology, University of Veterinary Medicine, 1078 Budapest, Hungary; farkas.robert@univet.hu (R.F.); lucarompos@gmail.com (L.R.); takacs.nora@univet.hu (N.T.); hornok.sandor@univet.hu (S.H.); 2 Department of Nature Conservation, Aggtelek National Park Directorate, 3758 Jósvafő, Hungary; sandorboldogh@yahoo.com; 3 ELKH-ÁTE Climate Change: New Blood-Sucking Parasites and Vector-Borne Pathogens Research Group, 1078 Budapest, Hungary; 4 Small Animal Clinic, 3780 Edelény, Hungary; beresnyakviktor@gmail.com; 5 Department of Ranger Service, 3758 Jósvafő, Hungary; izso.adam88@gmail.com; 6 Plant Protection Institute, Centre for Agricultural Research, ELKH, 1022 Budapest, Hungary; jkontschan@gmail.com; 7 Department of Plant Sciences, Albert Kázmér Faculty of Mosonmagyaróvár, Széchenyi István University, 9200 Mosonmagyaróvár, Hungary; 8 Fish and Conservation Ecology Research Group, Balaton Limnological Research Institute, ELKH, 8237 Tihany, Hungary; lanszkij@gmail.com

**Keywords:** feline, vector-borne, tick-borne, piroplasm, Hungary

## Abstract

Among vector-borne protozoa *Hepatozoon felis* and *Cytauxzoon europaeus* are considered emerging species in felids in Europe. To investigate the presence of these two protozoa 127 domestic cats and 4 wildcats were screened by PCRs targeting the *18S* rRNA gene of *Hepatozoon* spp. and piroplasms, as well as the *cytb* gene of *Cytauxzoon* spp. The samples were collected inside and outside a region of Hungary, where both protozoan groups are endemic in wildcats. Among domestic cats, one proved to be infected with *H. felis*. Furthermore, spleen samples of four wildcats were also examined, among which three tested positive for *H. felis*, and one had co-infection with *C. europaeus*. Importantly, *H. felis* from the co-infected wildcat belonged to genogroup II, similarly to *H. felis* from the positive domestic cat. Based on phylogenetic evidence, this genogroup probably represents a separate species from genogroup I of *H. felis*, which was hitherto reported from Mediterranean countries in Europe. The two other wildcats also harbored *H. felis* from genogroup I. Neither *Hepatozoon* nor *Cytauxzoon* infections were detected outside the recently discovered endemic region. In conclusion, this study demonstrates for the first time in Europe that *H. felis* from genogroup II may emerge in free-roaming domestic cats in regions where this protozoan parasite is endemic in wildcats.

## 1. Introduction

*Hepatozoon* (Adeleida: Hepatozoidae) and *Cytauxzoon* (Piroplasmida: Theileriidae) species are unicellular apicomplexan parasites with different modes of vector-borne transmission [1,2]. Namely, in case of the former, the main source of infection is the ingestion of the vector. However, in contrast to *Hepatozoon* spp., *Cytauxzoon* spp. infect the host by their tick vector during blood meal [3]. In the absence of vectors, transplacental infection is also thought to be possible in both of these apicomplexan groups [2,4,5].

In Europe, feline hepatozoonosis is caused by *Hepatozoon felis*, *Hepatozoon canis* and *Hepatozoon silvestris* [2,6]. Their vectors are unknown, however, *Rhipicephalus sanguineus* might be involved in the transmission of *H. felis* [2,7]. The development of *H. felis* takes place in skeletal and cardiac muscles of the host, and the gamont forms can be observed in neutrophil granulocytes during parasitemia, though, only less than 1% of the neutrophils are infected [5,8]. Therefore, it is recommended to use PCR for diagnosis [6]. Symptoms include anemia, increased levels of creatine-kinase (CK) and lactate-dehydrogenase (LDH), however, the infection often remains subclinical. Furthermore, there is no controlled treatment of feline hepatozoonosis, however, combination of imidocarb dipoprionate and doxycycline has proved to be effective [5]. In Europe the infection with *Hepatozoon* spp. can be considered emerging among domestic cats (*Felis catus*), since during the last decade several cases were reported in Western Europe [2] and in Mediterranean countries, e.g., in Italy, Spain and Greece [9,10,11,12]. In Central Europe the first clinical case in a domestic cat involving *H. felis* has recently been also described [13].

Feline cytauxzoonosis caused by *Cytauxzoon felis* is a long-recognized disease of domestic cats in North America, where the bobcat (*Lynx rufus*) is the natural reservoir of the infection [3]. The vectors are two tick species of the genera *Amblyomma* and *Dermacentor* [3,4]. The infection in domestic cats is often associated with high mortality and affected animals show fever, vomiting, anemia, icterus and hepatosplenomegaly. The detection of *C. felis* is frequently based on the examination of blood smears, however, molecular methods are more specific and sensitive in the identification [3,4,14]. Concerning the treatment of feline cytauxzoonosis the combination therapy of azithromycin and atovaquone (A&A) has proved to be the most effective, however, atovaquone targets the cytochrome *b* (*cytb*) and its efficacy is mutable [3].

In Europe, feline *Cytauxzoon* infection is caused by three recently described species, *Cytauxzoon europaeus*, *Cytauxzoon otrantorum* and *Cytauxzoon banethi* [15]. Their presence was reported in European wildcat (*Felis silvestris*) and lynx (*Lynx pardinus*, *Lynx lynx*), as well as in domestic cats from Switzerland, Germany, France, Italy, Spain and Portugal. Interestingly, European *Cytauxzoon* spp. seem less virulent than *C. felis* [16]. The vector has hitherto been unknown, but *Ixodes ricinus* or a *Dermacentor* sp. might play a role in the transmission [17]. In line with this, in Hungary *C. europaeus* was detected in *I. ricinus* removed from an infected wildcat [18].

The primary aim of the study was to screen for the presence of *H. felis* and *C. europaeus* in wildcats and outdoor domestic cats that live in a recently discovered endemic area in Hungary, where these infections were reported to emerge in wildcats [18]; furthermore, to examine domestic cats outside this endemic region in comparison.

## 2. Materials and Methods

**Figure 1:**
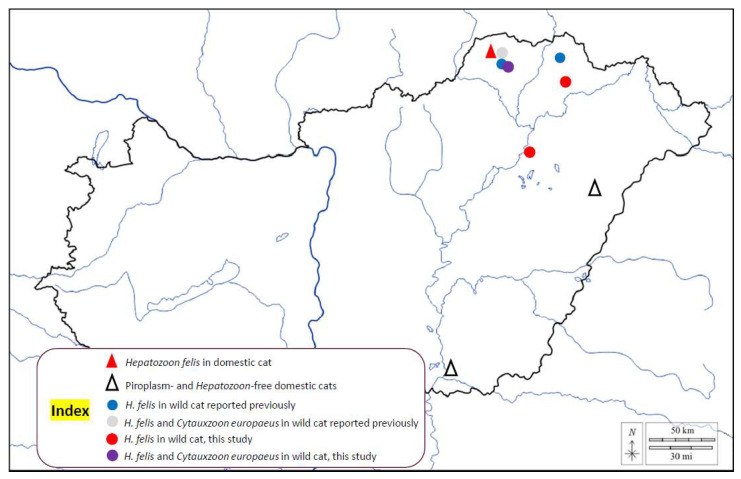
Map of Hungary showing the sampling sites and positive cases.

During this study, infections with *Hepatozoon* and *Cytauxzoon* spp. were investigated in 131 cats. In 2021–2022, anticoagulated (EDTA-containing) blood samples were collected from the *vena cephalica antebrachii* of 126 clinically normal domestic cats (*Felis catus*) with known history of outdoor activity: 88 were from non-endemic regions of eastern and southeastern Hungary (Debrecen: n = 73, Szeged: n = 15), and 38 from the recently discovered endemic region (north-eastern Hungary: Aggtelek National Park) (Figure 1). Blood smears were prepared from freshly collected blood samples taken from cats in the latter area, then stained with May–Grünwald–Giemsa and examined under light microscope (Leica Microsystems, Wetzlar, Germany). In addition, spleen samples of a domestic cat and four European wildcats (*Felis silvestris*) which were found as road-kills between 2015–2021 in the endemic area were also examined. All tissue was kept frozen at −20 °C until processing.

For DNA extraction from tissues, samples were taken from the middle of organs to exclude surface contamination, using a sterile scalpel blade or scissors. The DNA was extracted individually from 200 μL of collected blood or approximately 10 mg of spleen using the QIAamp DNA Mini Kit (Qiagen, Hilden, Germany) according to the manufacturer’s blood or tissue protocol. All DNA extracts were screened for piroplasms and *Hepatozoon* spp. The primers and cycling conditions of PCR analyses are summarized in Table 1 [19,20,21,22]. In the conventional PCR used to detect piroplasms (including *Cytauxzoon* spp.), 5 µL of extracted DNA was added to 20 µL of reaction mixture containing 1.0 U HotStar Taq Plus DNA Polymerase (5 U/µL) (QIAGEN, Hilden, Germany), 0.5 µL dNTP Mix (10 mM), 0.5 µL of each primer (50 µM), 2.5 µL of 10 × Coral Load PCR buffer (15 mM MgCl_2_ included) and 15.8 µL distilled water. In the PCR amplifying a fragment of the 18S rRNA gene of *Hepatozoon* spp. (approx. 650 bp), the protocol was modified by using 0.2 µL of each primer (50 µM), 1 µL extra MgCl_2_ (to a total of 25 mM) and 17.9 µL distilled water. Sequence-verified positive controls were included in all PCR analyses. Purification and sequencing of the positive PCR products were performed by Biomi Ltd. (Gödöllő, Hungary). The newly generated sequences were submitted to GenBank under accession numbers OQ102981 (18S rRNA gene of *H. felis* in domestic cat), OQ445870-OQ445872 (18S rRNA gene of *H. felis* in wildcat), OQ445869 (18S rRNA gene of *C. europaeus* in wildcat), OQ455050 (*cytb* gene of *C. europaeus* in wildcat). Sequences were aligned and compared to reference GenBank sequences by nucleotide BLASTn program (https://blast.ncbi.nlm.nih.gov (accessed on 3 March 2023)).

For the phylogenetic analysis, we calculated 1000 bootstrap replicates. The tree was generated with the Maximum Likelihood method and Jukes–Cantor model after performing model search by the program MEGA version 7.0 [23].

## 3. Results

**Figure 2:**
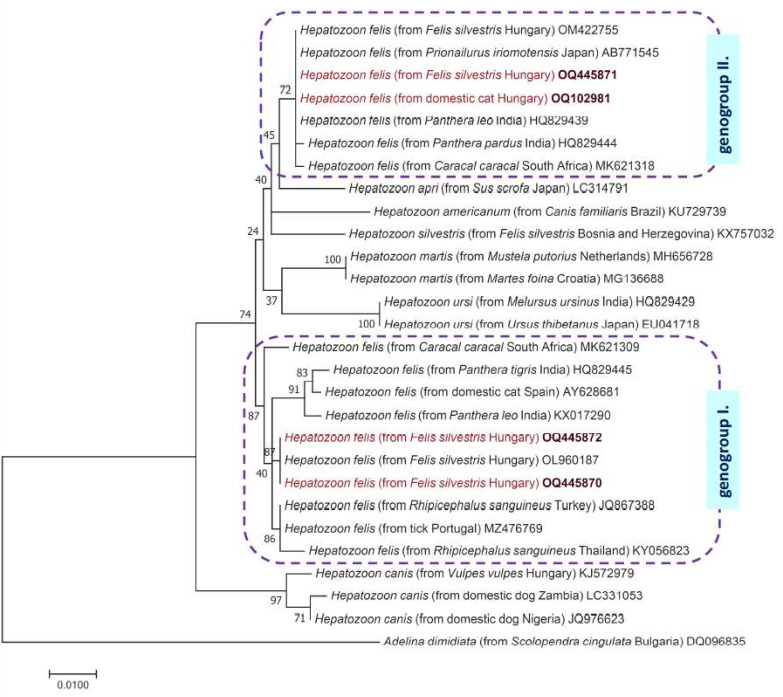
Phylogenetic tree of *Hepatozoon* species from carnivores based on *18S* rRNA gene sequences. In each row, after the species name, the host species, country of origin, and the GenBank accession number are shown. New sequences (from this study) are marked with red fonts and maroon accession numbers. The evolutionary history was inferred by using the Maximum Likelihood method and the Jukes–Cantor model. The tree with the highest log likelihood is shown. The percentage of trees in which the associated taxa clustered together is shown next to the branches. The tree is drawn to scale, with branch lengths measured in the number of substitutions per site. The analysis involved 28 nucleotide sequences, and there were a total of 581 positions in the final dataset. *Adelina dimidiata* (Apicomplexa: Adeleidae) was used as outgroup. All positions containing gaps and missing data were eliminated.

Concerning domestic cats in the study, only 1 out of 127 proved to be positive in the PCR detecting *Hepatozoon* spp., and this cat was kept in the region where *H. felis* is endemic among wildcats (Figure 1). The corresponding 18S rRNA sequence (OQ102981) showed 100% (596/596 bp) identity with *H. felis* from *F. silvestris* in Hungary (OM422755) and 99.8% (622/623 bp) sequence identity to another isolate reported from a leopard in India (OL852083). Importantly, the *Hepatozoon* sequence identified for the first time in a domestic cat of the endemic region belonged to genogroup II of *H. felis* (Figure 2). However, in the blood smear of the affected cat, no gamonts were seen in neutrophil granulocytes. None of the domestic cats were PCR positive for piroplasms, including *Cytauxzoon* spp.

Three of the four wildcats examined in this study tested positive for *H. felis*, among which one had co-infection with *C. europaeus* (Supplementary Table S1). In wildcats that had mono-infection with *H. felis* one sequence (OQ445871) showed 100% (593/593 bp) identity to *H. felis* from *F. silvestris* in Hungary (OM422755), i.e., from genogroup II as the sample from the domestic cat (see above). However, two further samples (OQ445870, OQ445872) were 100% (595/595 bp) identical to a sequence (OL960187) reported previously from wildcats in Hungary representing genogroup I of *H. felis*. The phylogenetic relationships of these sequences are shown in Figure 2.

Furthermore, one *H. felis*-infected wildcat was PCR-positive for piroplasms. The 18S rRNA gene sequence from this cat (OQ445869) showed 100% (452/452 bp) identity with *C. europaeus* from a wildcat in Germany (ON380465). At the same time, the corresponding *cytb* sequence had only 99.6% (1183/1188 bp) identity with *C. europaeus* from a wildcat also from Germany (ON856000), and was also different (only 1187/1188 bp identical) from the single conspecific sequence reported previously in Hungary.

## 4. Discussion

In this study, the occurrence of *H. felis* and *C. europaeus* was investigated primarily in domestic cats in an area, where both protozoan parasites are endemic in wildcats [18]. The European wildcat is distributed widely in Europe, from the Iberian Peninsula to the Caucasus Mountains. The spread and genetic diversity of the species are threatened by the closely related domestic cat, since free-range domestic cats might be in a much higher density in their natural habitat, which creates the conditions for hybridization and close contact as well as sharing pathogens [24]. This has been reported at a high level in Hungary [25]. In addition, those cats with outdoor lifestyle are exposed to different arthropods, such as fleas and ticks, which may transmit different vector-borne pathogens [26] including *Hepatozoon* spp. and piroplasms. Furthermore, with the increase of urbanization and common living space the role of different reservoirs, such as lynx and wildcat in the sylvatic cycle increases the possibility of pathogen transmission to domestic cats [27].

Feline hepatozoonosis is assumed to be transmitted by ticks, e.g., *R. sanguineus*, presumably similarly to *H. canis* [7]. Feline cytauxzoonosis is known to be a tick-borne disease, although in Europe the tick vector needs clarification [17].

In a worldwide context, similarly to our result, the same *18S* lineage of *H. felis* had been found in an Asiatic lion (*Panthera leo persica*) in India (HQ829439) [28]. Thereafter, the same “genogroup II” lineage of *H. felis* was also reported in an Iriomote cat (*Prionailurus iriomotensis*) in Japan (AB771519) [29]. In Europe, *H. felis* is indigenous almost exclusively to Mediterranean countries, where it has been emerging in domestic cats since 2017. In a study 7% of domestic cats imported from the Mediterranean or South-Eastern Europe to Germany were positive to *H. felis* [2]. In Southern Italy 5.1% of domestic cats were positive with PCR and they were infected mainly with *H. felis*, however, one of them was infected with *H. canis*, and another one with *H. silvestris* [9]. Interestingly, few years later in North-Eastern Italy, *H. silvestris* was found with a higher prevalence [11]. In other Mediterranean countries, Spain and Greece, *H. felis* was reported with 1.6% and 25.5% prevalence, respectively [10,12]. As an exception among non-Mediterranean countries, in Austria an autochthonous case was reported, affecting a six-year-old tick-infested male cat which showed symptoms of anorexia, fever, icterus and was diagnosed with hepatozoonosis [13]. It is important to note that *H. felis* reported from Mediterranean countries in Europe represents genogroup I (e.g., under AY628681 from Spain or KY649442 from Italy) which is probably a different species than *H. felis* of genogroup II, detected in domestic cats in Central Europe in this study, as shown by the phylogenetic analysis (Figure 2).

During our investigation in Hungary, one domestic cat proved to be infected with *H. felis*. However, this cat did not show any clinical signs of the disease, and no gamonts were found in its neutrophil granulocytes. This might be due to the low parasitemia, i.e., usually only 1% of neutrophils are infected [5]. It is very important to note that the *H. felis*-infected domestic cat was kept outdoors in the Aggtelek National Park, where this protozoan parasite has recently been recognized to cause emerging infection among wildcats [18]. This is the only area in Europe north of the Mediterranean Basin where *H. felis* is known to be endemic. No cats were PCR positive outside this region in Hungary.

The present study describes the first evidence of the presence of *H. felis* in domestic cats in Hungary. In a broader context, this is the first evidence in Europe that domestic cats can become infected with *H. felis* from genogroup II where this species is endemic in wildcats. Although different wild rodents may also take part in its transmission [30], the infection most likely happened by ingesting infected ticks in the habitat (wooded area of Aggtelek National Park) shared with wildcats.

Feline cytauxzoonosis is considered a severe disease in the United States, with acute course and high mortality [3]. Nevertheless, in Europe *Cytauxzoon* spp. might be less virulent species, which mainly cause subclinical infection, or clinical manifestation associated with co-infection or immunodeficiency [17]. In Europe several countries (France, Italy, Germany and Switzerland) reported the presence of *Cytauxzoon* sp. in domestic cats without [11] or with clinical signs [16,17,31,32]. Moreover, in Portugal a *Cytauxzoon* sp.-infected cat was described as showing clinical symptoms without co-infection with FIV or FeLV, and eventually the cat died [33]. Thus, feline cytauxzoonoosis in Europe may even have a serious outcome. Investigating the domestic cats in our study, none of them had PCR-positive result, but among wildcats one was infected with *C. europaeus* in co-infection with *H. felis*. *Cytauxzoon europaeus* has been reported in wildcats in different European countries, i.e., in Germany, the Czech Republic, Luxembourg, Bosnia and Herzegovina, Italy, Switzerland, France and Hungary [16,18].

In conclusion, feline hepatozoonosis and cytauxzoonosis are emerging infections in the southern part of Central Europe. Hitherto *H. felis* and *C. europaeus* have only been found in wildcats in this endemic area, but according to the present results at least *H. felis* also emerged in domestic cat. This study suggests for the first time in Europe that *H. felis* from genogroup II may emerge in free-roaming domestic cats in such regions where this protozoan parasite is endemic in wildcats. The prevalence of *H. felis*-infection in this region was shown to be relatively high among wildcats, both in our previous and this study, but (based on the present results) is low among domestic cats. To monitor the possible emergence of *H. felis* with higher prevalence or in other populations of domestic cats and the transmission of *C. europaeus* from wild to domestic cats in the region, further investigations are needed.

## Figures and Tables

**Table 1:** Primers and details for conventional PCR methods used in this study.

Target Group	Target Gene	Primer Name	Primer Sequence (5′-3′)	Amplicon Length (bp)	Thermocycling Profile	Reference
**Piroplasms**	18S rRNA	BJ1	GTC TTG TAA TTG GAA TGA TGG	500	95 °C for 10 min; 40× (95 °C for 30 s; 54 °C for 30 s; 72 °C for 40 s); 72 °C for 5 min	[19]
		BN2	TAG TTT ATG GTT AGG ACT ACG			
***Hepatozoon* spp.**	18S rRNA	HepF	ATA CAT GAG CAA AAT CTC AAC	650	95 °C for 5 min; 35× (95 °C for 40 s; 57 °C for 40 s; 72 °C for 60 s); 72 °C for 7 min	[20]
		HepR	CTT ATT ATT CCA TGC TGC AG			
***Cytauxzoon* spp.**	*cytb*	Cytaux_cytb_Finn	ACC TAC TAA ACC TTA TTC AAG CRT T	1333	95 °C for 5 min; 45× (95 °C for 20 s; 55 °C for 30 s; 68 °C for 1.5 min); 68 °C for 7 min	[21,22]
		Cytaux_cytb_Rinn	AGA CTC TTA GsAT GYA AAC TTC CC			

## Data Availability

Not applicable.

## Supplementary Materials

The following supporting information can be downloaded at: https://www.mdpi.com/article/10.3390/pathogens12050656/s1, Table S1: Data and PCR results of domestic and wild cats from the Aggtelek National Park.
