# Supplementary material for: Screening of Domestic Cats from North-Eastern Hungary for Hepatozoon felis and Cytauxzoon europaeus That Cause Infections in Local Wildcat Populations
Source: Pathogens. 2023 Apr 28;12(5):656. doi: 10.3390/pathogens12050656 (PMC10221201; doi:10.3390/pathogens12050656)
Supplement: Supplementary file 1 [file pathogens-12-00656-s001.zip › pathogens-2338309-supplementary/Supplementary Table S1.pdf]

Supplementary Table S1. Data and PCR results of domestic and wild cats from the Aggtelek National Park.

Sample ID	Sample Source	Sex	Location	PCR Result *Hepatozoon/Cytauxzoon*
**WC21**	***Felis silvestris***	**male**	**Bodrogolaszi**	+/-
**WC22**	***Felis silvestris***	**male**	**Újszentmargita**	+/-
WC23	*Felis silvestris*	male	Múcsony	-/-
**WC24**	***Felis silvestris***	**male**	**Szendrő**	+/+
WC25	*Felis catus*	male	Ónod	-/-
AGCAT1	*Felis catus*	male	Jósvafő	-/-
AGCAT2	*Felis catus*	female	Jósvafő	-/-
AGCAT3	*Felis catus*	male	Jósvafő	-/-
AGCAT4	*Felis catus*	male	Jósvafő	-/-
AGCAT5	*Felis catus*	female	Jósvafő	-/-
AGCAT6	*Felis catus*	male	Jósvafő	-/-
AGCAT7	*Felis catus*	female	Jósvafő	-/-
AGCAT8	*Felis catus*	female	Jósvafő	-/-
AGCAT9	*Felis catus*	male	Jósvafő	-/-
AGCAT10	*Felis catus*	female	Jósvafő	-/-
AGCAT11	*Felis catus*	male	Jósvafő	-/-
AGCAT12	*Felis catus*	male	Jósvafő	-/-
AGCAT13	*Felis catus*	male	Jósvafő	-/-
AGCAT14	*Felis catus*	female	Komjáti	-/-
AGCAT15	*Felis catus*	female	Komjáti	-/-
AGCAT16	*Felis catus*	female	Komjáti	-/-
AGCAT17	*Felis catus*	female	Komjáti	-/-
AGCAT18	*Felis catus*	male	Komjáti	-/-
AGCAT19	*Felis catus*	female	Komjáti	-/-
AGCAT20	*Felis catus*	male	Szögliget	-/-
**AGCAT21**	***Felis catus***	**female**	**Szinpetri**	+/-
AGCAT22	*Felis catus*	female	Jósvafő	-/-
AGCAT23	*Felis catus*	female	Szinpetri	-/-
AGCAT25	*Felis catus*	female	Szinpetri	-/-
AGCAT26	*Felis catus*	female	Szinpetri	-/-
AGCAT27	*Felis catus*	male	Szinpetri	-/-
AGCAT28	*Felis catus*	male	Szendrő	-/-
AGCAT29	*Felis catus*	female	Szendrő	-/-
AGCAT30	*Felis catus*	female	Szendrő	-/-
AGCAT31	*Felis catus*	female (neutered)	Komjáti	-/-
AGCAT32	*Felis catus*	female (neutered)	Komjáti	-/-
AGCAT33	*Felis catus*	female (neutered)	Komjáti	-/-
AGCAT34	*Felis catus*	male (neutered)	Komjáti	-/-
AGCAT35	*Felis catus*	male (neutered)	Komjáti	-/-
AGCAT36	*Felis catus*	female (neutered)	Edelény	-/-
AGCAT37	*Felis catus*	female (neutered)	Szendrő	-/-
AGCAT38	*Felis catus*	male (neutered)	Felsőnyárád	-/-
AGCAT39	*Felis catus*	female (neutered)	Felsőnyárád	-/-
